# Enhanced model protein adsorption of nanoparticulate hydroxyapatite thin films on silk sericin and fibroin surfaces

**DOI:** 10.1007/s10856-021-06632-5

**Published:** 2021-12-23

**Authors:** Selçuk Özcan, Muhsin Çiftçioğlu

**Affiliations:** 1 Department of Industrial Engineering, Bilecik Şeyh Edebali University, Bilecik, Turkey; 2 Department of Chemical Engineering, Izmir Institute of Technology, Urla, Izmir, Turkey

## Abstract

Hydroxyapatite coated metallic implants favorably combine the required biocompatibility with the mechanical properties. As an alternative to the industrial coating method of plasma spraying with inherently potential deleterious effects, sol-gel methods have attracted much attention. In this study, the effects of intermediate silk fibroin and silk sericin layers on the protein adsorption capacity of hydroxyapatite films formed by a particulate sol-gel method were determined experimentally. The preparation of the layered silk protein/hydroxyapatite structures on glass substrates, and the effects of the underlying silk proteins on the topography of the hydroxyapatite coatings were described. The topography of the hydroxyapatite layer fabricated on the silk sericin was such that the hydroxyapatite particles were oriented forming an oriented crystalline surface. The model protein (bovine serum albumin) adsorption increased to 2.62 µg/cm^2^ on the latter surface as compared to 1.37 µg/cm^2^ of hydroxyapatite on glass without an intermediate silk sericin layer.

**Figure:**
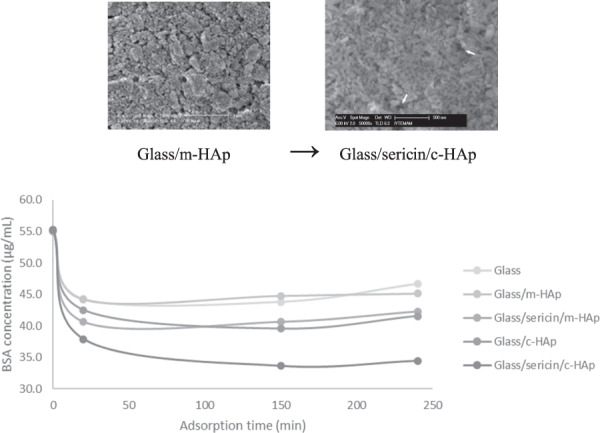
The BSA adsorption on glass (blank), glass/c-HAp, glass/m-HAp, glass/sericin/c-HAp, and glass/sericin/m-HAp substrates, reported as decrease in BSA concentration versus contact time.

The BSA adsorption on glass (blank), glass/c-HAp, glass/m-HAp, glass/sericin/c-HAp, and glass/sericin/m-HAp substrates, reported as decrease in BSA concentration versus contact time.

## Introduction

The formation of hydroxyapatite in vivo as the mineral constituent of bone and teeth tissue is closely related to the physiology of calcium and phosphate metabolism in vertebrates. The intricate hormonal control (parathyroid hormone and calcitonin with the combined effect of vitamin D_3_) mechanisms of osteoblastic and osteoclastic activities, including the feedback control of calcium and phosphate extracellular fluid levels, regulate the deposition and resorption of calcium phosphate (CaP) compounds in the bone [1]. The embodiment of hard tissue implants to the host bone tissue is achieved through morphological fixation, osteoconduction, osseointegration, chemical adhesion, or time regulated resorption, with healing accelerated osteoinductively, governed by the closely related biological and chemical processes. The intended permanent or transitory anchorage of the implant in the living tissue with a minimal foreign body reaction, for proper biofunctioning, and without premature failure, is engineered by material development, design, and strict quality assured manufacturing [2].

Biocompatible mineral coatings of CaP allow a variety of materials to be employed as in vivo hard tissue implants [3, 4]. They act as local scaffolds for enhanced osteoconduction by osteogenic cell migration, proliferation, and differentiation on the implant surface (osteoinduction) resulting in ingrowth of the surrounding hard tissue. Hydroxyapatite (HAp), Ca_10_(PO_4_)_6_(OH)_2_, has been experimentally confirmed to be one of the best implant coating materials for post-operative fast bone apposition and cementless mechanical fixation based on the concerted dynamic resorption-precipitation-bone substitution in vivo processes [5]. Plasma spraying of HAp is the only method commercially approved by the U.S. Food and Drug Administration (FDA) for biomedical coatings on implants so far [6]. However, there is a general tendency to develop new routes for hydroxyapatite coatings on implant materials due to the fact that coatings produced by the plasma spraying have a number of disadvantages, namely, inhomogeneous chemical composition and crystallinity, formation of in vivo fast dissolving unfavorable phases (α-tricalcium phosphate, tetracalcium phosphate, oxyhydroxyapatite, calcium oxide, and amorphous phases), thermal and amorphous to crystalline phase transformation stress cracking, and delamination or even detachment of the coating layer [7–10]. There exist active research efforts to overcome the adverse effects [11, 12]. Hydroxyapatite film coatings prepared by sol-gel methods have attracted much attention due to the inherent advantages of being chemically and physically homogeneous and pure, capability of tailoring chemical composition, porosity, pore size and surface roughness, besides the relative simplicity of the method [13–15]. Nevertheless, some of the HAp coatings achieved by the sol-gel techniques, especially, when HAp forms in-situ during the coating process, yields an amorphous or poorly crystalline HAp layer that may suffer the drawbacks due to the amorphous HAp phase as in plasma spraying [6].

The favorable interactions between hydroxyapatite, and silk fibroin and sericin in the biomimetic nucleation and crystal growth of hydroxyapatite deposits, were experimentally observed [16, 17]. The aspartate, arginine, glutamate residues are arranged in polar clusters in the β-sheet structure of fibroin (characterized by the repetitive primary sequence of amino acids of a long chain of 350 kDa, connected to a shorter chain by disulfide linkage [18]), inducing a surface potential distribution due to the hydroxyl, carboxyl, and carbonyl groups, that have an affinity for calcium ions, providing local concentrations of Ca^2+^ leading to nucleation of HAp crystals. HAp is deposited in the form of tiny crystals on fibroin due to the regular surface arrangement of nucleation sites distributed on a molecular scale. In the β-sheet structure of sericin (molecular weight 20–400 kDa [19]), 10% of the carboxyl groups are arranged perpendicular to the plane of the sheet, providing a periodic distribution of surface electrostatic potential sites which induces electrostatic affinity for Ca^2+^ ions leading to HAp nucleation and crystal growth.

Fracture or peri-implant healing involves interactions between the critical physical, molecular, and cellular elements inducing the sequence of events: potentially osteogenic cell recruitment, attachment, expansion, differentiation, and bone tissue generation. Cytokines and bone morphogenetic proteins (BMPs) from the disrupted matrix, and from degranulation of platelets in the hemorrhage zone signal and guide the marrow derived osteogenic cell populations including vascular pericytes, stromal cells and mesenchymal stem cells (MSCs) to migrate to the damaged site. MSCs are converted to preosteogenitor cells on the site which then transform into osteoblastic, chondroblastic, or fibroblastic differentiated cell lineages. The technology to use CaP compound coatings as BMPs carrier is under development by imparting enhanced affinity between hydroxyapatite and proteins [2, 20]. In many of these studies bovine serum albumin (BSA) was used as model protein to mimic the behavior of BMPs [21]. The surface topography of the hydroxyapatite was shown to be as important as the chemistry for protein adsorption [22, 23].

In this study, hydroxyapatite thin film implant surfaces were prepared by a particulate sol route. A variety of particle dispersion techniques were used to prepare HAp colloidal suspensions. Preparation of thin films of HAp with the colloidal suspensions on bioinert glass surfaces by the dip coating method was investigated. The microstructure of the films was characterized by scanning electron microscope (SEM), atomic force microscope (AFM), x-ray diffraction (XRD) and Fourier transform infra-red spectroscopy (FTIR), while the results for the latter two were presented elsewhere [24]. The particulate sol-gel method utilizing the commercially pre-synthesized fully crystalline HAp powder made sure that the HAp layer was crystalline and remained crystalline after the heat treatment [24]. The effects of the intermediate silk fibroin and silk sericin coatings on the formation and surface topography of HAp films were determined. The model protein (BSA) adsorption capabilities of the various hydroxyapatite surfaces were investigated comparatively.

## Experimental

### The hydroxyapatite powder size reduction

The commercially available hydroxyapatite powder (c-HAp; Ca_10_(PO4)_6_(OH)_2_, SIGMA, C 567–500 G, FW = 1004.6, mp = 1670 °C, d = 3.14 g/cm^3^) was used for thin film preparations, as received, and after dry ball milling in a 250 ml tungsten-carbide jar revolved in a gyrator. The grinding medium comprised 30 pieces tungsten-carbide balls of 1 cm in diameter. Each load for milling consisted of 15 g of c-HAp. The milling was carried out for a maximum of 120 min at 400 rpm with intermittent 15 min grinding and cooling periods (from this point on the 120 min dry milled powder will be denoted as m-HAp powder; m for dry milled). The details of the particle size distribution measurements and the methods for dispersion in deionized water were given elsewhere [24]. Shortly, the particle size distribution measurements were carried out with the X-ray scattering sedigraph (Micromeritics Sedigraph 5100), and the measurement concentration of the suspensions was 0.02 g/mL (1.0 g of solids was dispersed in 50 ml with deionized water). c-HAp powder was dispersed in deionized water by various methods (and also combinations of methods) at various solids loadings with hydropalate (2 w/w%; sodium di-isooctylsulfosuccinate, C_20_H_38_O_7_S, MW = 422.58, Henkel) as dispersant. The dispersion methods were ultrasound treatment, wet ball milling, fast mixing in baffled blender, suspension quenching in liquid nitrogen, thermal shock by water quenching of heated powder.

### The pH, and organic dispersant ratio

The optimum dispersion of c-HAp, and m-HAp powder suspensions were determined by the zeta sizer (Malvern Instruments Ltd. 3000 HSA). Stock suspensions of c-HAp and m-HAp powders were prepared by adding 10 g of powder into 47 g of deionized water. The organic dispersant, hydropalate 64, was added at various amounts (0–0.36 g or 6 drops). The deagglomeration was carried out by wet ball milling in glass jars with a grinding medium of zirconia balls (30 g of Ø5mm, and 15 g of Ø3mm) for 2 h at 200 rpm. The stock suspension was then diluted to a solid content convenient for measurement in the zeta sizer. 0.2 ml of the stock solution was first diluted to 10 ml. The diluted suspension was ultrasonicated for 30 min, and 1.0 ml of it was diluted to 20 ml in a beaker that corresponds to an overall 1000-fold dilution. The final solid content was approximately 0.17 g/L. Before the measurements, the pH of the suspension in the beaker was adjusted in the range from 7 to 11 by 0.1 N NH_4_OH addition, and ultrasonicated for an additional 15 min while stirring.

### Preparation of hydroxyapatite/silk protein thin films by dip coating

Thin film coatings of hydroxyapatite were achieved by dip coating of bioinert glass slide substrates in dispersed suspensions. The glass slides were cleaned by detergent solution, ethyl alcohol, and acetone subsequently. 15 w/w% aqueous suspensions of c-HAp, and m-HAp powders were prepared in deionized water. The steric dispersant hydropalate 64 was added in the ratio 2.4 w% of the total solids. The pH of the c-HAp, and m-HAp suspensions were adjusted to 9.8–10.0, and 9.4–9.6 (with 1.0 N NH_4_OH), respectively. These 50 ml suspensions were ultrasonically treated (30 kHz) while stirring for 30 min, followed by wet ball milling in 100 ml jars with a grinding medium of zirconia balls (30 g of Ø5 mm, and 15 g of Ø3 mm) for 2 h at 200 rpm. Finally, the suspensions were ultrasonically treated for 2 hr before dip coating.

The bioinert glass substrates were dip coated in the c-HAp and m-HAp suspensions at 25 °C with a retraction rate of 100 mm/min. The substrates were coated up to 5 layers with 30 min of ambient drying in between. Some of the thin films on glass substrates were heat treated at 560 °C, for 30 min with a heating ramp rate of 15 °C/min. The film thicknesses were measured with Mitutoyo SJ-301 surface roughness tester in the primary profile mode, generating a length-depth profile of the film scratched with a 85 shore A hard rubber.

The sericin and fibrion film coatings on glass substrates were prepared by dip coating in the sericin and fibroin solutions with a retraction rate of 100 mm/min at ambient temperature (25 °C). The preparations of sericin and fibroin solutions are described below. The substrates were coated with three layers of sericin and fibroin, with 30 min of ambient drying between coatings. The sericin and fibroin coated glass slides were than coated with c-HAp and m-HAp in the same way described above.

The sericin solution was prepared by dissolving 4.2 g of commercial sericin (Silk Biochemical Co. Ltd., ref: 46-3-108) in 48 ml of deionized water. Silk fibroin solutions were prepared by the subsequent processes of degumming and dissolution of the raw silk (Silk Biochemical Co. Ltd., ref no: biosilk 04056). The degumming was carried out by boiling in 0.05% aqueous Na_2_CO_3_ solution (2.0 g of silk in 100 mL solution) for 30 min. The degummed silk was washed with deionized water and left over for drying at ambient conditions. 1.2 g of the degummed silk was further treated with 20 times of Ajisawa’s reagent (24 mL of CaCl_2_/ethanol/water: 111/92/144 by weight) in a 250 mL Schott bottle by stirring at 78 °C for 2 h to obtain a clear solution [25]. Higher concentrations were avoided due to the gelation tendency during the following dialysis step. The fibroin solution was dialyzed in cellulose dialysis tubing against deionized water at 4 °C by frequent change of dialysis water for the removal of CaCl_2_ and other neutral salts until tested negative for AgCl precipitation by AgNO_3_ (~3 days). The treated cellulose tubings were rated to retain protein molecules of over 12,000 g/mol. The silk fibroin solution (1–2% w/v) in the dialysis tubings was filtered (Filtrak 389 filter paper) and concentrated to ~10% w/v by a rotary vacuum evaporator at 30 °C, 30 rpm.

### Bovine serum albumin and collagen adsorption on thin films

The adsorption behavior of bovine serum albumin (BSA, the adsorbate) on the hydroxyapatite thin films (c-HAp, and m-HAp) was investigated. The quantity of BSA adsorbed on the HAp surfaces was determined as a function of contact time. The adsorbent substrates were bioinert glass, glass/c-HAp, glass/m-HAp, glass/sericin/c-HAp, and glass/sericin/m-HAp.

The bovine serum albumin (BSA; SIGMA CAS 9048-46-8, A2153-100G, Albumin from bovine serum, min. 96%, electrophoresis) solution was prepared in 100 mM PBS (phosphate buffer saline). 50 mg/L solution was prepared and diluted in two steps to 0.050 mg/mL (50 μg/mL). All dilutions were carried out by 100 mM PBS solution.

The adsorption was carried out in 100 mL beakers in the incubator kept at 37 °C, and 60 rpm. 5 substrates were placed in each beaker to form 59.8 cm^2^ adsorption area. The BSA concentration was measured as a function of contact time. The BSA concentration was measured by an HPLC size exclusion method. The column used was ZORBAX bio series GF-250 (LX14004 PN 884973.901 max pressure 350 bar). The mobile phase used was 100 mM PBS solution. The UV-vis detector module set at 210 nm wavelength. The 50 μL of the BSA solution sample was manually injected. Two peaks were obtained for BSA; the small peak belonged to the dimer of BSA, and a major peak with the retention times of 4.55 min and 4.96 min, respectively. The results were reported as the corresponding total area of the two peaks. The calibration curve was formed for a range of concentrations from 0.01 to 0.10 mg/L.

The SEM images of the thin films were obtained by Philips XL-30S FEG. For AFM imaging (Digital Instruments MMSPM Nanoscope IV was used in the tapping mode with rectangular silicon nitride cantilever, and silicon tip to obtain the 3-dimensional surface topology, and phase information of the film surfaces), BSA solution, and collagen type I aqueous suspension were prepared in 10 mM PBS (phosphate buffer saline). The concentration of BSA was adjusted to 1.0 mg/mL (total volume 25 ml). Collagen type I (SIGMA C-9879 1 g, Type I, Insoluble, from Bovine Achilles Tendon, 9007-34-5) was water insoluble, and it was dispersed as a homogeneous suspension in ultrasound bath. The concentration was 0.1 mg/mL (total volume 25 ml). The glass, glass/sericin, glass/fibroin, glass/c-HAp, glass/m-HAp, glass/sericin/c-HAp, glass/sericin/m-HAp, glass/fibroin/c-HAp, glass/fibroin/m-HAp substrates were prepared by dip coating as described previously, and the samples were cut into maximum 1 × 1 cm size suitable for AFM measurements. Two samples of each type of coating were placed separately in the BSA solution and the collagen type I suspensions perpendicularly to avoid any gravitational settling on the films. The samples were kept in the suspensions for 4 hr. The samples were rinsed with deionized water to sweep away the loosely bound BSA particles and collagen fibers and left over for ambient drying.

## Results

### Particulate sol route hydroxyapatite/silk protein thin film surface interactions

**Fig. 1:**
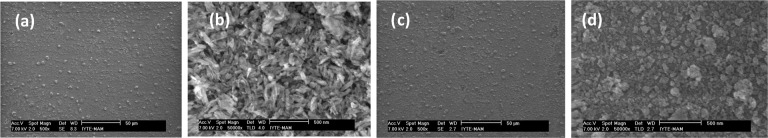
The SEM images of the c-HAp coatings on the glass substrates prepared by 15w% solid content sol by dip coating, at various magnifications (**a**) 500x, (**b**) 50,000x, and the m-HAp coatings on the glass substrates prepared by 15w% solid content sol by dip coating at various magnifications (**c**) 500x, (**d**) 50,000x.

**Fig. 2:**
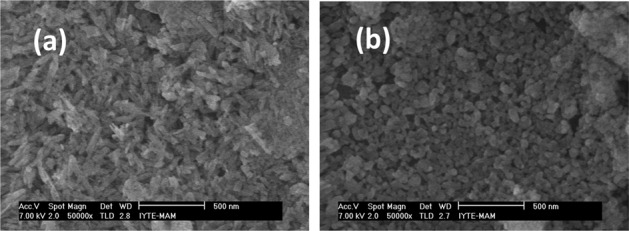
The SEM images of the thin films prepared with 15w% HAp suspensions after the heat treatment at 560 °C, for 30 min. with a heating rate of 15 °C/min at various magnifications (**a**) c-HAp 50,000x, (**b**) m-HAp 50,000x.

As a result of the dry ball milling the tapped density increased from 0.44 g/cm^3^ (c-HAp) to 0.96 g/cm^3^ (m-HAp). The commercial powder consisted of rod like particles of 150–250 nm in length and 50–80 nm in diameter, while the dry milled powder consisted of equiaxed particles of 40–100 nm in diameter. The details of hydroxyapatite powder characterization, dispersion, and coating were given elsewhere [24]. The coatings of HAp were prepared on bioinert glass substrates by dip coating the SEM pictures of which as can be seen in Fig. 1. The aqueous suspensions of HAp contained 2.4w% hydropalate 64 as the dispersant, and at pH values of 10.2 for c-HAp and 9.4 for m-HAp, with 15w% solids content of the suspensions. The maximum agglomerate sizes were determined to be 2 μm for the coatings of c-HAp, and 5 μm for the coatings of the m-HAp. The agglomerates of the sizes from 450 nm to 1 μm were most abundant for both type of coatings. The agglomerates larger in number and size in the m-HAp coatings, proved to be an indication of the higher agglomeration tendency, during the drying/particle compaction step of the thin film formation, with increasing surface area, and hence, lower zeta potential of the crushed particles in the suspensions [24]. The thicknesses of the thin films prepared with the c-HAp powder were determined to be in the range 450–650 nm, while the ones with the m-HAp were in the range 500–750 nm. The effect of heat treatment, at 560 °C, for 30 min with a heating ramp rate of 15 °C/min, on the thin films prepared was investigated. There were no cracks forming de novo, or propagating. Nevertheless, the onset of sintering, particles fusing together, could be seen in Fig. 2a, b for c-HAp and m-HAp coatings, respectively.

XRD and FTIR were employed for the characterization of the thin films. The thin film grazing incidence XRD patterns of the c-HAp and m-HAp coatings before, and after the heat treatment at 560 °C for 30 min can be found elsewhere [24]. The patterns were identified as hydroxyapatite, the peaks of which superimposed on the broad peak that belonged to the amorphous glass substrate. The major peaks in all of the patterns were at 2θ values of 32.9, 32.2, 31.8, 28.9, 25.9 degrees. The relative intensities of the peaks of c-HAp films at 2θ = 32.9, 32.2, 31.8 degrees were lower, in comparison to m-HAp films. The relative peak intensities of the heat treated (560 °C, 30 min) films were slightly higher as compared to their counterparts, which might have been due to crystal size increase. However, heat treatment had no detectable effects on the crystallinity of the hydroxyapatite films, and there were not any phase changes, as can be expected. The chemistry of the c-HAp and the m-HAp surfaces were thus taken as identical [22].

**Fig. 3:**
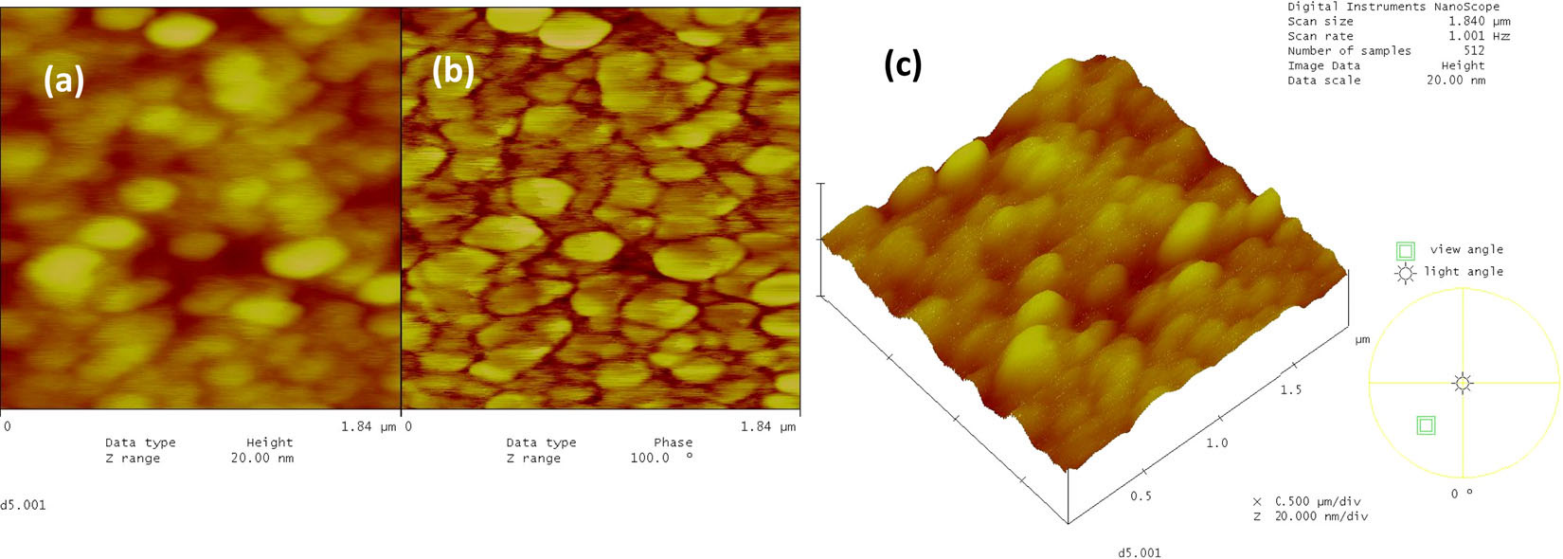
The AFM images of the sericin film on glass substrate, (**a**) 2-dim. topographical, (**b**) phase, and (**c**) 3-dim. topographical images, 1.84 × 1.84 μm.

The topographical AFM image for the sericin film on the glass substrate (Fig. 3) showed a granular total surface coverage with the average granular size being in the range of 150–250 nm, and sporadic agglomerates in the range of 400–600 nm. The phase images indicated a homogeneous surface with insignificant agglomeration. This phenomenon is most likely due to an isotropic structure exhibiting thickness differences as can be seen in Fig. 3c.

**Fig. 4:**
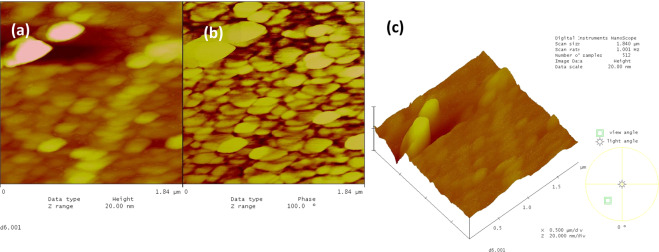
The AFM images of the fibroin film on glass substrate, (**a**) 2-dim. topographical, (**b**) phase, and (**c**) 3-dim. topographical images, 1.84 × 1.84 μm.

The AFM image for the fibroin film on glass substrate (Fig. 4) showed that the surface was totally covered with fibroin and with discernable grains of sizes 100–200 nm. The grains tend to align in straight lines reminiscent of fibrous structures. The topographical image showed sporadic lumps of fibroin agglomerates in the range of 400–600 nm while the phase image did not indicate such agglomeration. This again most probably points out to a fluctuating thickness of an isotropic film (Fig. 4c).

These sericin and fibroin coated glasses were used as substrates for the dip coating of 15w% solid content suspensions of c-HAp, and m-HAp particulate sols. The minimum threshold solid content of suspensions in order to obtain full surface coverage on the sericin and fibroin layers, was determined to be 15w%. The films coated on the glass/sericin substrates were totally transparent, while the films coated on the glass/fibroin substrates were slightly opacified.

**Fig. 5:**
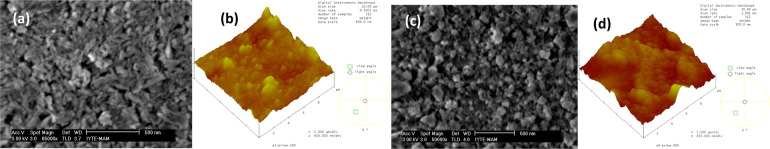
The SEM and AFM images of the c-HAp and m-HAp films (with 15w% solid content suspension) on the glass/sericin substrate (**a**) the SEM image of c-HAp film, 65,000x, (**b**) the AFM 3-dim. topographical image of c-HAp film, 10 × 10 μm, (**c**) the SEM image of m-HAp film, 50,000x (**d**) the AFM 3-dim. topographical image of m-HAp film, 10 × 10 μm.

The SEM and AFM images of the c-HAp and m-HAp films on glass/sericin substrates are shown in Fig. 5. The c-HAp films were decisively more homogeneous as compared to their counterparts coated on the glass substrates. The agglomerate sizes were also less than 300 nm explaining the visible light transparency of the films. The surfaces were predominantly covered by primary particles, and the thickness of the HAp layer on top of the sericin layer was measured to be 150–200 nm (determined by profile measurements), indicating a film of 3–4 layers of primary particles compaction. The AFM image showed the same homogeneous feature of the films. The agglomerate like structures in the topographical images were most probably due to the fluctuating thickness differences of the intermediate sericin film. The SEM and AFM images of the m-HAp films on glass/sericin substrates again showed that the films were decisively more homogeneous as compared to their counterparts coated on glass substrates. However, the surfaces were covered predominantly by agglomerates, evidencing the increased agglomeration tendency of the higher surface area particles. The films were still visible light transparent since the agglomerate sizes were less than 300 nm. The agglomerates consisted of primary particles with sizes less than 50 nm. The thickness of the HAp layer on top of the sericin layer was measured as 150–200 nm. The AFM image also showed agglomerates as in the SEM images. The highly fluctuating thickness of the sericin film beneath was responsible of the lumps in the topographical image.

**Fig. 6:**
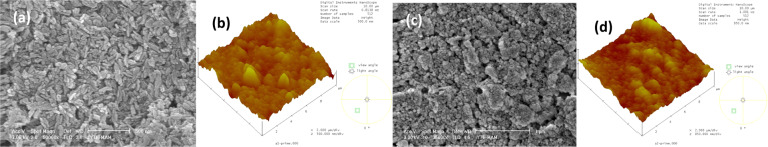
The SEM and AFM images of the c-HAp and m-HAp films (with 15w% solid content suspension) on the glass/fibroin substrate (**a**) the SEM image of c-HAp film, 50,000x, (**b**) the AFM 3-dim. topographical image of c-HAp film, 10 × 10 μm, (**c**) the SEM image of m-HAp film, 35,000x (**d**) the AFM 3-dim. topographical image of m-HAp film, 10 × 10 μm.

The SEM and AFM images of the c-HAp films on glass/fibroin substrates are shown in Fig. 6. The films were more homogeneous as compared to their counterparts coated on glass substrates. However, the agglomerate sizes were in the range 400 nm - 1 μm, and covered ~35% of the surface, partially reflracting light as well as scattering, which explains the slightly opacified appearance of the films. The thickness of the c-HAp layer on top of the fibroin layer was measured to be 150–200 nm. This again corresponds to a film of 3–4 layers of packed primary particles. The AFM topographic image indicated the same particle and agglomerate features of the films. The thickness fluctuation of the fibroin films (1–2 μm) were not so extensive as in the case of sericin films, however, the effect was still visible with the agglomerate like structures in the topographical images. The SEM and AFM images of the m-HAp films on glass/fibroin substrates showed that again the films were more homogeneous as compared to their counterparts coated on glass substrates. The maximum agglomerate size was close to 2 μm, and predominantly the agglomerate sizes were less than 500 nm. The films were again slightly opacified. The thickness of the m-HAp layer on top of the fibroin layer was measured to be 250–600 nm. The AFM topographical image also showed agglomeration with agglomerates dominant in the range 100–500 nm, as in the SEM images. The effect of the variable thickness of the intermediate fibroin film was seen in the topographical images.

**Fig. 7:**
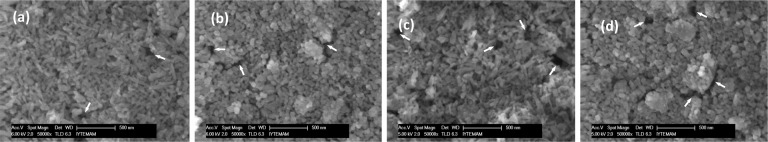
SEM images of (**a**) the glass/sericin/c-HAp, (**b**) the glass/sericin/m-HAp, (**c**) the glass/fibroin/c-HAp, (**d**) the glass/fibroin/m -HAp thin film structures after heat treatment at 560 °C; the arrows indicate some of the hole-like gaps through which sericin and fibroin burnt out.

The glass/sericin/c-HAp, glass/sericin/m-HAp, glass/fibroin/c-HAp and glass/fibroin/m-HAp structures were heat treated at 560 °C, for 30 min with a heating ramp rate of 15 °C/min, and the effect is shown in Fig. 7. The hydroxyapatite coatings remained intact, and the onset of sintering was apparent especially for the coatings on the sericin layer. The hole-like gaps (some of which are indicated by the arrows) were formed most probably because of the gaseous products evolution during the burning of sericin and fibroin layers.

Apparently, the effect of intermediate sericin and fibroin films was the selective deposition of particles of minimal available sizes, predominantly of the primary particles. The affinity of the spatially recurring carboxyl groups (­COO¯) in the β-sheet structure of silk proteins, for Ca^2+^ of the hydroxyapatite crystal was reported in the literature; the other possibility that amino groups, -NH_3_^+^ or =NH_2_^+^, to have an affinity for PO_4_^3−^ in the hydroxyapatite crystal was considered as a minor effect in comparison to the former because of the polar concentrated arrangement (rather than regular spatial arrangement) of these groups in the β-sheet protein structure [16–18, 26].

The forces in effect for the hydroxyapatite particle deposition on the silk sericin and fibroin films were the same as adsorption phenomenon. By that token the HAp particle deposition during the thin film formation process was most likely due to the coulombic interaction between the regular arrangement of surface negative charge distribution of sericin and fibroin in the β-sheet structure provided by the ­COO¯ groups, and the recurring positive surface charge site strips rich in Ca^2+^ on the (100) and (010) faces of hexagonal HAp crystals. On the (001) face of the HAp crystal lattice, 6 oxygen ions that belong to three crystal phosphates create negative charge sites, which may have a coulombic attraction for the polar –NH_3_^+^ groups in the β-sheet structure. The former coulombic interaction probably provided a partially positive degree of cooperativity for the HAp particle deposition on the sericin and fibroin surfaces; together with the latter coulombic interaction the degree of cooperativity might change from partially positive to positive [27]. However, the agglomeration tendency between the HAp particles was a factor that reduced the degree of cooperativity.

The thin coatings of essentially primary particles of HAp, with drastically reduced number and size of agglomerates, and homogeneity on the sericin, and the fibroin films were thus resulted from the surface energy minimization of the deposition process driven by coulombic attraction, and probably pointed out to a high degree of positive cooperativity. The agglomeration tendency of the smaller size HAp particles deteriorated the degree of cooperativity, and hence, the surface homogeneity which was visible in the glass/sericin/m-HAp, and especially glass/fibroin/m-HAp coatings shown in Figs. 5c and 6c, respectively. The coatings with the c-HAp, namely, glass/sericin/c-HAp and glass/fibroin/c-HAp in Figs. 5a and 6a, respectively, indicated a lower tendency for agglomeration and a more homogeneous film structure, which was most probably the result of a higher degree of positive cooperativity in the deposition.

On the other hand, the HAp coatings on sericin were more homogeneous with less agglomerates of smaller sizes in comparison to the coatings on fibroin as can be seen in Fig. 5a, b, and Fig. 6a, b. This might be due to a more pronounced random coil structure with respect to β-sheet structure in the fibroin films, and due to an inherent surface charge distribution even in the β-sheet structure of fibroin reducing the degree of positive cooperativity for HAp deposition. The surface charge distribution regularities are expected to be delimited by the surface topography, and crystal size and shape which were also factors in determining the HAp particle deposition on the sericin and fibroin surfaces.

### Enhanced model protein adsorption of silk protein/hydroxyapatite thin films

BSA was used to model the adsorption behavior of bone growth factors or cytokines on the hydroxyapatite coated surfaces. The affinities of BSA, a globular protein, and collagen, a fibrillar protein for the aforementioned film surfaces were investigated by AFM imaging. The effect of an intermediate silk sericin thin layer beneath the hydroxyapatite coatings, on the BSA adsorption, was quantified.

**Fig. 8:**
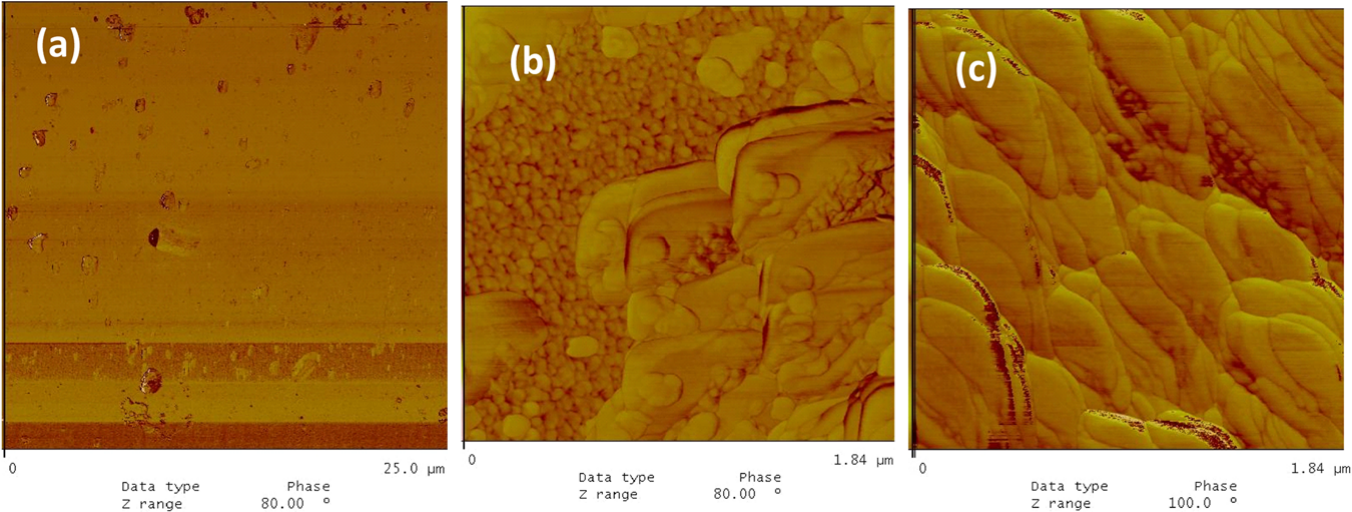
The AFM phase images of adsorbed BSA on (**a**) glass, (**b**) glass/c-HAp film (**c**) glass/m-HAp film.

The AFM phase image of the BSA adsorbed on glass is given in Fig. 8a. The glass surface was virtually featureless, and the BSA particles were discernable in both the topological and the phase images as equiaxed particles in accord with the fact that BSA particles are globular [28]. Few BSA particles occupied the glass surface. The AFM phase images of BSA adsorbed on glass/c-HAp and glass/m-HAp films substrates are shown in Fig. 8b, c, respectively. The 1.84 × 1.84 μm images illustrated the adsorption of BSA particles of the approximate size range of 20–50 nm. The adsorbed BSA quantity on the glass/m-HAp film was nearly the same as on the glass. However, the quantity adsorbed on the glass/c-HAp film was higher than the previous substrates.

**Fig. 9:**
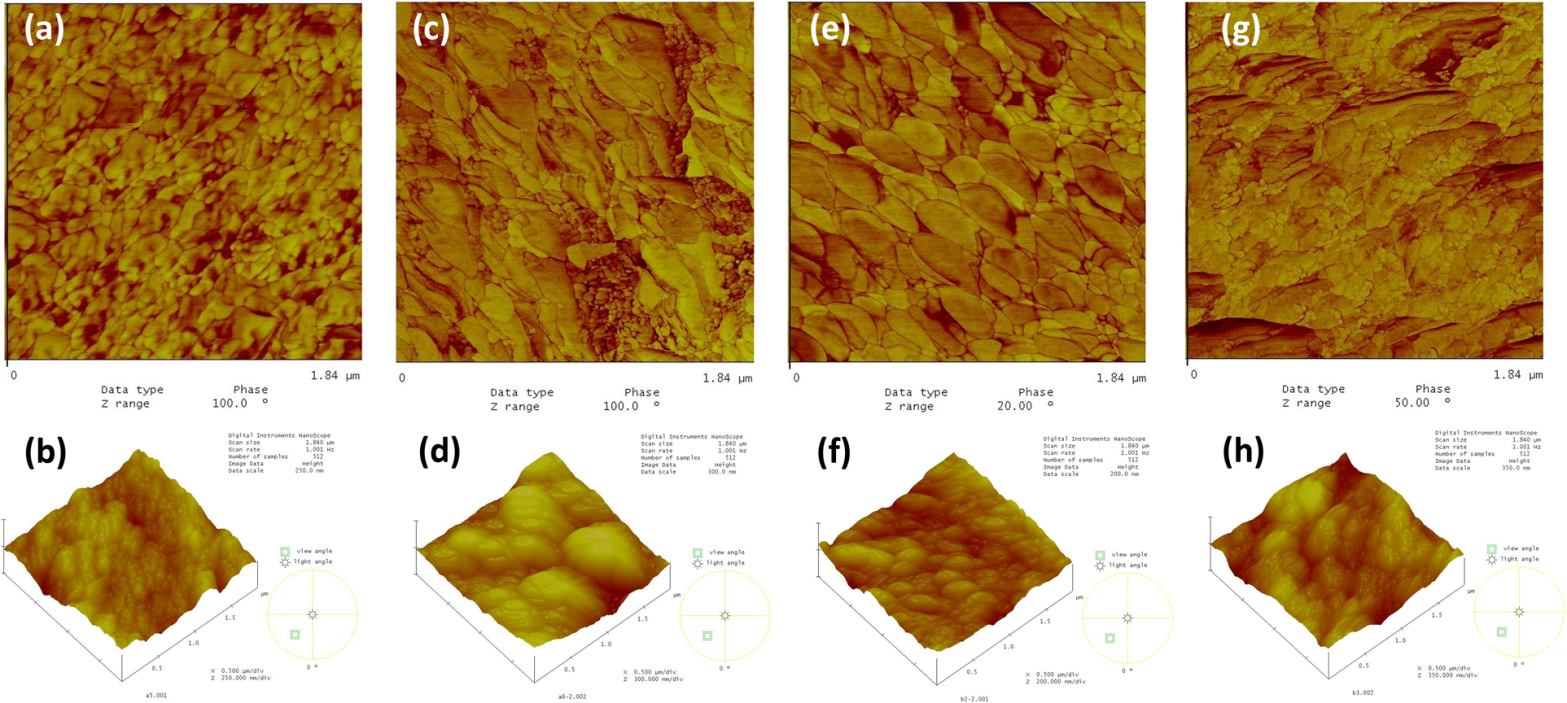
The AFM images of adsorbed BSA on (**a**) the glass/fibroin/c-HAp; phase image, (**b**) the glass/fibroin/c-HAp; 3-dim. topographical image, (**c**) the glass/fibroin/m-HAp; phase image, (**d**) the glass/fibroin/m-HAp; 3-dim. topographical image, (**e**) the glass/sericin/c-HAp; phase image, (**f**) the glass/sericin/c-HAp; 3-dim. topographical image, (**g**) the glass/sericin/m-HAp films; phase image, (**h**) the glass/sericin/m-HAp films; 3-dim. topographical image.

The AFM phase images of the BSA adsorption on fibroin/c-HAp, fibroin/m-HAp, sericin/c-HAp, and sericin/m-HAp films are shown in Fig. 9. The 1.84 × 1.84 μm phase image, and 1.84 × 1.84 μm 3-dimensional topographical image scans provided the insight. From higher to lower, the adsorption capacities of the films were ranged as sericin/c-HAp, fibroin/c-HAp, sericin/m-HAp, and fibroin/m-HAp. The discernable BSA phase indicated that glass/sericin/c-HAp and glass/fibroin/c-HAp film structures adsorbed multilayer of BSA particles which was drastically higher than the BSA adsorbed on the glass/sericin/m-HAp and glass/fibroin/m-HAp films, as well as the glass/c-HAp and glass/m-HAp films. On the fibroin/c-HAp film the BSA particles covered the HAp surface with agglomerates of 50–300 nm, while on the fibroin/m-HAp film the surface coverage comprised BSA particles of the size 50 nm on the average. The multilayer BSA layer adsorbed on the sericin/c-HAp surface consisted of the BSA particle agglomerates of the sizes ranged in 50–400 nm. The sericin/m-HAp film surface coverage of the BSA was of agglomerate sizes of 35–50 nm.

The glass/sericin/c-HAp provided the best coating for BSA adsorption. The glass/fibroin/c-HAp proved to be the next best for the BSA adsorption. The glass/sericin/m-HAp coating proved to be slightly better than its glass /fibroin/m-HAp counterpart for adsorbing BSA. However, the BSA adsorption affinity was drastically lower with the m-HAp films over sericin and fibroin surfaces. This indicated that the adsorbed BSA quantity reduced on the smaller particle size HAp films, glass/m-HAp, glass/fibroin/m-HAp and glass/sericin/m-HAp alike, most probably due to the extensive HAp particle agglomeration disrupting the regular surface charge distribution. The positive degree of cooperativity in the coulombic attraction driven adsorption process was therefore reduced. The adsorption process of protein molecules was affected by the topographical structure of the surface as well as the chemical structure.

**Fig. 10:**
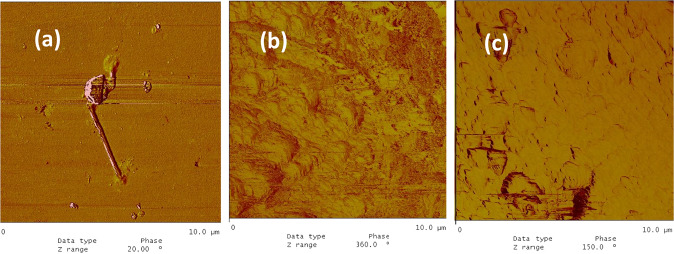
The AFM phase image of adsorbed collagen type I on (**a**) the glass, (**b**) the glass/c-HAp film, (**c**) the glass/m-HAp film.

The AFM image of the collagen type I adsorbed on the glass surface is shown in Fig. 10a. The adsorption was negligible. The AFM images of the collagen type I adsorption on c-HAp, and m-HAp films on glass substrates, are given in Fig. 10b, c, respectively. Although on the c-HAp film collagen fibers were very little, on the m-HAp surface there were not any collagen fibers observed.

**Fig. 11:**
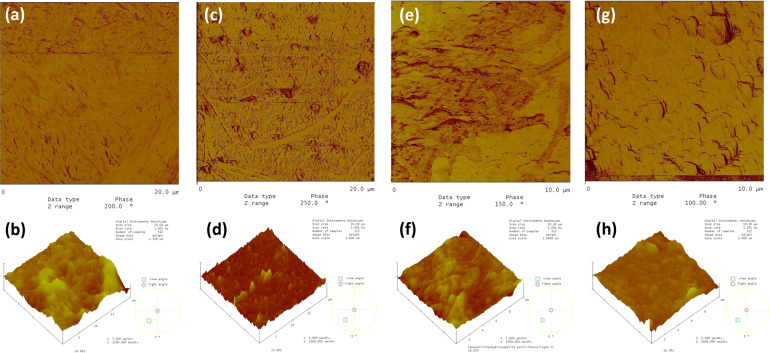
The AFM images of adsorbed collagen type I on (**a**). **a** the glass/fibroin/c-HAp; phase image, (**b**) the glass/fibroin/c-HAp; 3-dim. topographical image, (**c**) the glass/fibroin/m-HAp; phase image, (**d**) the glass/fibroin/m-HAp; 3-dim. topographical image, (**e**) the glass/sericin/c-HAp; phase image, (**f**) the glass/sericin/c-HAp; 3-dim. topographical image, (**g**) the glass/sericin/m-HAp films; phase image, (**h**) the glass/sericin/m-HAp films; 3-dim. topographical image.

The collagen type I adsorption on the glass/fibroin/c-HAp and glass/fibroin/m-HAp films are given in Fig. 11a, b, respectively. On the c-HAp film the collagen fiber adsorption was extensive with nearly full surface coverage, while on the m-HAp film the surface coverage was very little. The collagen type I adsorption on the glass/sericin/c-HAp, and glass/sericin/m-HAp films are given in Fig. 11c, d, respectively. The collagen coverage of the c-HAp film was roughly half of the surface, evidencing an affinity less than for the glass/fibroin/c-HAp coating. On the m-HAp surface there were no observable collagen fiber adsorption at all. However, near the edge of the film where glass substrate was exposed directly to the collagen suspension little collagen fiber was observed.

These results for the collagen type I fibers indicated that surface topographic structure proves to be an important factor, as well as the chemical structure, determining adsorption affinity. The coatings with the intermediate fibroin layers provided a better adsorption affinity for the collagen type I fibers as compared to the coatings with the intermediate sericin layer, a phenomenon which was reverse for that of the BSA adsorption.

The c-HAp particles might have been spatially distributed over the fibroin surface during their deposition, forming a charge distribution complementary and corresponding to that of the fibroin film underneath which might be considered as a template. Therefore, the collagen adsorption was also enhanced on the glass/fibroin/c-HAp coating, because of the matching surface charge of the c-HAp layer distributed in accordance with the fibroin film template. This phenomenon also pointed out that the interaction between the fibroin film and collagen fibers was predominantly coulombic rather than hydrophobic, since the adsorption also enhanced on the c-HAp surface with the intermediate fibroin film acting as a template. The collagen type I fiber adsorptions on the m-HAp surfaces were much lower (or even non-existent) as compared to the c-HAp surfaces.

### Quantification of the adsorption of BSA on the thin films of c-HAp and m-HAp on glass, and glass/sericin substrates

**Fig. 12:**
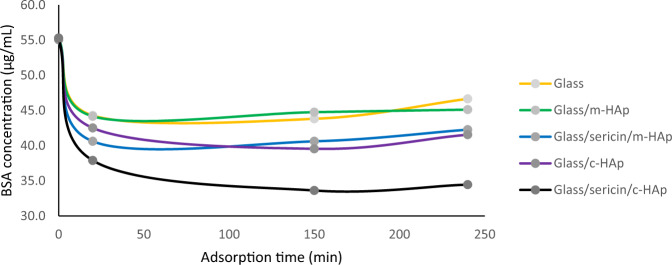
The BSA adsorption on glass (blank), glass/c-HAp, glass/m-HAp, glass/sericin/c-HAp, and glass/sericin/m-HAp substrates, reported as decrease in BSA concentration versus contact time.

The BSA concentrations were measured by the HPLC size exclusion method, with 100 mM PBS solution as the mobile phase. The decrease in the BSA concentration was monitored as a function of time. The R^2^ equals 0.9915 for the calibration curve obtained for the total BSA including its dimmer. The BSA adsorption on glass, glass/c-HAp, glass/m-HAp, glass/sericin/c-HAp, glass/sericin/m-HAp was studied. The change in BSA solution concentration with time is given in Fig. 12. The glass substrates adsorbed BSA as well. The glass/m-HAp film did not show any enhanced adsorption over glass substrates. The BSA adsorption of glass/sericin/m-HAp substrate was nearly the same as glass/c-HAp substrate. The BSA concentration at 150 min was ~9% lower in comparison to glass substrate (a decrease from 43.8 μg/mL for glass to 39.6 μg/mL for glass/c-HAp). The largest decrease in BSA concentration was for the glass/sericin/c-HAp film. The difference from glass substrate was around 30% (43.8 μg/mL for glass, 33.6 μg/mL for glass/sericin/c-HAp film) at 150 min. Therefore, the adsorption of BSA on glass/sericin/c-HAp film was the highest, a phenomenon supported by the AFM imaging results. Another feature of the adsorption curve was in that the time rate of concentration was fast until 20 min after which it slowed down or even flattened.

**Fig. 13:**
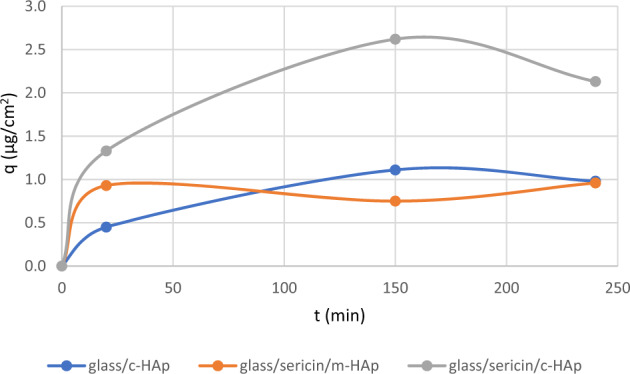
BSA adsorbed quantity as a function of time on the glass/c-HAp, the glass/sericin/m-HAp, and the glass/sericin/c-HAp films.

The adsorbed quantities per unit area are given in Fig. 13. For the glass/c-HAp and glass/sericin/m-HAp films, the maximum adsorption was around 1 μg/cm^2^ and there was a tendency for the adsorbed amount to decrease at elongated times. The maximum adsorption of BSA was on glass/sericin/c-HAp film, 2.6 μg/cm^2^, a result evidencing the effectiveness of surface topographical structure as well as the chemical structure for BSA adsorption on hydroxyapatite surfaces.

## Discussion

The purpose of this work was to prepare hydroxyapatite/silk protein thin film implant surfaces by a particulate sol-gel method, and to investigate their microstructural properties and model protein interactions for enhanced osteoinduction. By the SEM and AFM images, the minimum solid ratio of the suspensions was determined to be 15% for complete surface coverage of the glass substrates coated with c-HAp powder suspension; with the same solid ratio ~90% of the substrate surfaces were coated with the m-HAp powder suspension. The c-HAp films on glass/sericin substrates were decisively more homogeneous as compared to their counterparts coated on glass substrates. Approximately 85% of the surface was covered with primary particles, and the agglomerate sizes were also less than 300 nm explaining the visible light transparency of the films. The thickness of the c-HAp layer on top of the sericin layer was measured to be 150–200 nm. The m-HAp films on glass/sericin substrates were also more homogeneous as compared to glass substrates. The surface was covered predominantly by agglomerates, however, less than 400 nm in size, evidencing the increased agglomeration tendency of the higher surface area particles, compared to c-HAp. Still the films were visible light transparent. The thickness of the m-HAp layer on top of the sericin layer was 150–200 nm. For both the c-HAp and the m-HAp films on glass/fibroin substrates the films were more homogeneous compared to glass substrates, and slightly scattered light with the predominant aggregate sizes of 400–500 nm; the maximum aggregate sizes reached 1–2 μm. The thickness of the c-HAp layer on glass/fibroin substrates were 150–200 nm, while m-HAp layer on the glass/fibroin substrate was 250–600 nm.

Apparently, the effect of intermediate sericin and fibroin films was the selective deposition of primary HAp particles with a certain crystal orientation. The regular arrangement of the carboxyl groups protruding from the β-sheet surfaces of the silk proteins provided a surface potential distribution that electrostatically bound Ca^2+^. The regularity was expected to be delimited by the surface topography, and crystal size and shape could also be factors in determining the particle packing on the surface. The high affinity of the silk proteins for BSA and collagen type I were confirmed by AFM imaging. The glass/sericin/c-HAp provided the best layered film for BSA adsorption. Glass/fibroin/c-HAp proved to be the next best for the BSA adsorption. The sericin film underlying the c-HAp proved to be slightly better than its fibroin counterpart for adsorbing BSA. However, the BSA adsorption affinity was found to be drastically lower with the m-HAp films over sericin and fibroin surfaces. The BSA adsorption was quantified by the HPLC analysis. For the glass/c-HAp and glass/sericin/m-HAp films, the maximum adsorption was around 1 μg/cm^2^. The maximum adsorption of BSA was on glass/sericin/c-HAp film, 2.6 μg/cm^2^, a 2.5 fold increase over unoriented crystal films, a result evidencing the effectiveness of surface topographical structure as well as the chemical structure [29]. Likewise, collagen type I adsorption was drastically lower on the glass/sericin/m-HAp and glass/fibroin/m-HAp films compared to glass/sericin/c-HAp and glass/fibroin/c-HAp films. Again, indicating the surface topography to be effective on the protein adsorption affinity of the HAp films.

A regular surface charge distribution of the adsorbent (HAp) induces cooperative adsorption of the flexible BSA molecule [27]. The SEM and AFM images of the HAp coatings indicated much less agglomeration on the intermediate sericin films. The agglomeration is a factor that increases surface roughness and disrupts the regularity of surface charge zones, which in turn reduces the degree of cooperativity for coulombic attraction driven adsorption. The flexible structure, and charged side groups of BSA, together with the regular surface charge strips of the adsorbent HAp surface most probably provided a positive degree of cooperativity in adsorption and the adsorbed quantity of BSA increased when the surface topography was facilitating. This phenomenon was supported by the work of Santos, et al. [22], in which the nano topography of calcium phosphates (HAp, and β-TCP) strongly affected the protein adsorption (serum albumin), being more important than the surface chemistry. Similar results were obtained by them showing that albumin adsorption increased ~5 fold on a surface with a roughness of 32 ± 6 nm in comparison to a rougher surface of identical chemistry with surface roughness 142 ± 24 nm [22]. The charge distributions and surface topographical structures of the adsorbate and the adsorbent have to match for enhanced adsorption. A particularly oriented crystalline hydroxyapatite surface was also reported to assist certain biological functions [30, 31].

The first structure that is formed during peri-implant healing is the cementline which biochemically requires adsorbed growth factors on the implant surface, and mechanically requires a stable surface with micron scale roughness or porosity with nanoscale undercuts, reinforced with collagen fibers with which it can interdigitate and interlock for a strong adhesion. The subsequent anchorage and growth of bone tissue on the cementline is also accelerated by the availability of the growth factors on the healing site [5]. Specifically synthesized hydroxyapatite was studied for drug loading and delivery [32]. It was shown that layered structures prepared such as TiO_2_/fluoro-hydroxyapatite/hydroxyapatite increased osteoblast cell proliferation in-vitro for orthopedic and dental implantation [33], and efforts existed to fabricate coatings for controlled drug delivery for faster healing and patient comfort, by sol-gel methods [34]. In this study, it was shown that c-HAp films on the intermediate fibroin and sericin layers had enhanced adsorption affinity for the collagen type I and BSA, respectively. This indicates that such structures may greatly enhance the in vivo adsorption of collagen fibrils and growth factors on the implant surfaces and provide a very convenient surface at the peri-implant healing site for osteoinduction and osteoconduction, facilitating the formation of an initial cementline interdigitating and interlocking with the implant surface and the subsequent development of an anchored bone tissue. Also, for accelerated healing the implant surfaces can be loaded with the convenient growth factors before implantation for in vivo delivery of these proteins.

## Conclusions

The high affinity of the silk proteins for BSA and collagen type I were confirmed by AFM imaging. The glass/sericin/c-HAp provided the best layered film for BSA adsorption. Glass/fibroin/c-HAp proved to be the next best for the BSA adsorption. The sericin film underlying the c-HAp proved to be slightly better than its fibroin counterpart for adsorbing BSA. However, the BSA adsorption affinity was found to be drastically lower with the m-HAp films over sericin and fibroin surfaces. The BSA adsorption was quantified by the HPLC analysis. For the glass/m-HAp and glass/sericin/m-HAp films, the maximum adsorption was around 1 μg/cm^2^. The maximum adsorption of BSA was on glass/sericin/c-HAp film, 2.6 μg/cm^2^, a result evidencing the effectiveness of surface topographical structure as well as the chemical structure. Likewise, collagen type I adsorption was drastically lower on the glass/sericin/m-HAp and glass/fibroin/m-HAp films compared to glass/sericin/c-HAp and glass/fibroin/c-HAp films. Again, indicating the surface topography to be effective on the protein adsorption affinity of the HAp films. The study implies that if bone morphogenetic proteins (BMPs) are loaded prior to implantation, or by the ability of the coated implant surface to adsorb the growth factors and cytokines in vivo, this will signal the migration, attachment, and differentiation of the pluripotent stem cells accelerating the peri implant healing process.
